# Supplementary material for: The Escherichia coli chromosome moves to the replisome
Source: Nat Commun. 2024 Jul 17;15:6018. doi: 10.1038/s41467-024-50047-z (PMC11255300; doi:10.1038/s41467-024-50047-z)
Supplement: Supplementary file 1 — Supplementary Information [file 41467_2024_50047_MOESM1_ESM.pdf]

Figures S1-S11

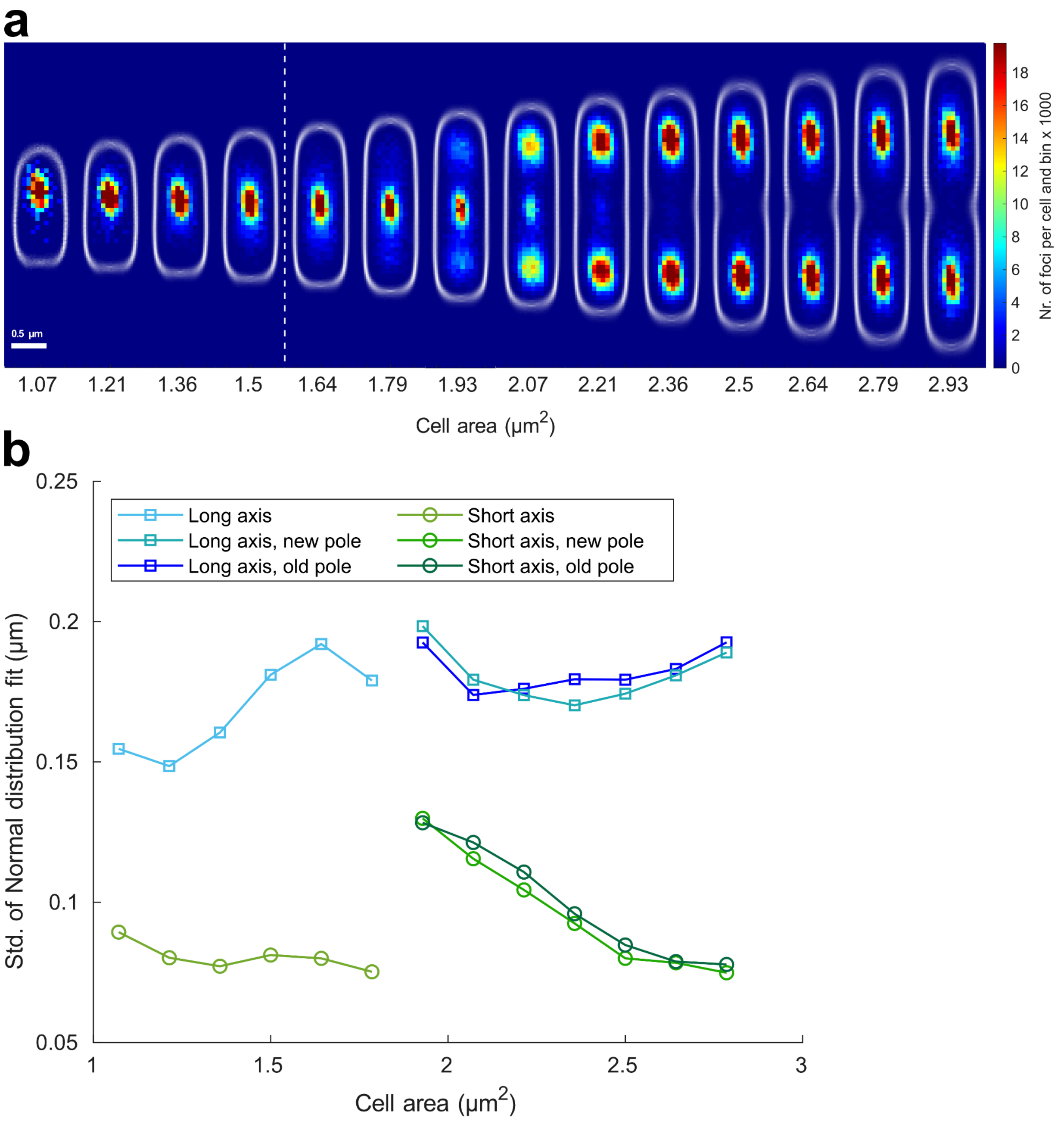

Fig. S1: Width of the replisome (YPet-DnaN) localization distributions at different cell sizes. a, As in Fig.1, but with YPet-DnaN as a replisome marker. **b,** Each peak of the replisome location distributions as shown in a, was fitted to a 2D uncorrelated normal distribution multiplied by a constant. The fitted standard deviations of the Normal distributions for the same cell size bins as of Fig. 1 are plotted for both the cell long axis (squares) and the cell short axis (rings).

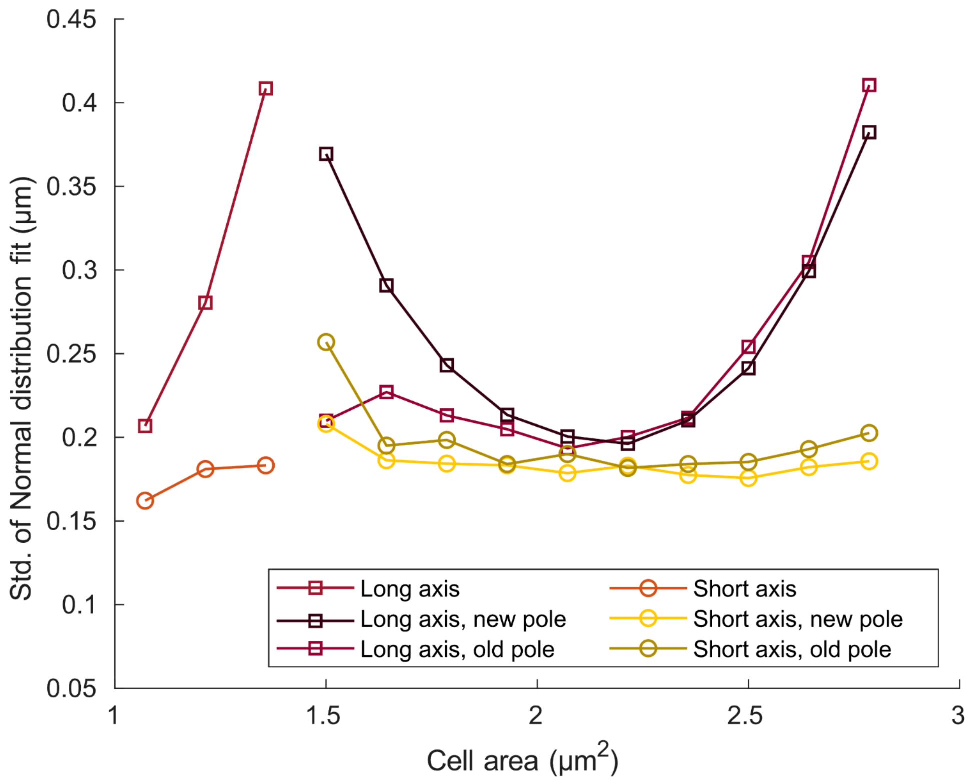

Fig. S2: Width of the *oriC* localization distributions at different cell sizes. As in Supplementary Fig. S1, but for the *oriC*-proximal label, with fitting based on the location distributions shown in Fig. 1.

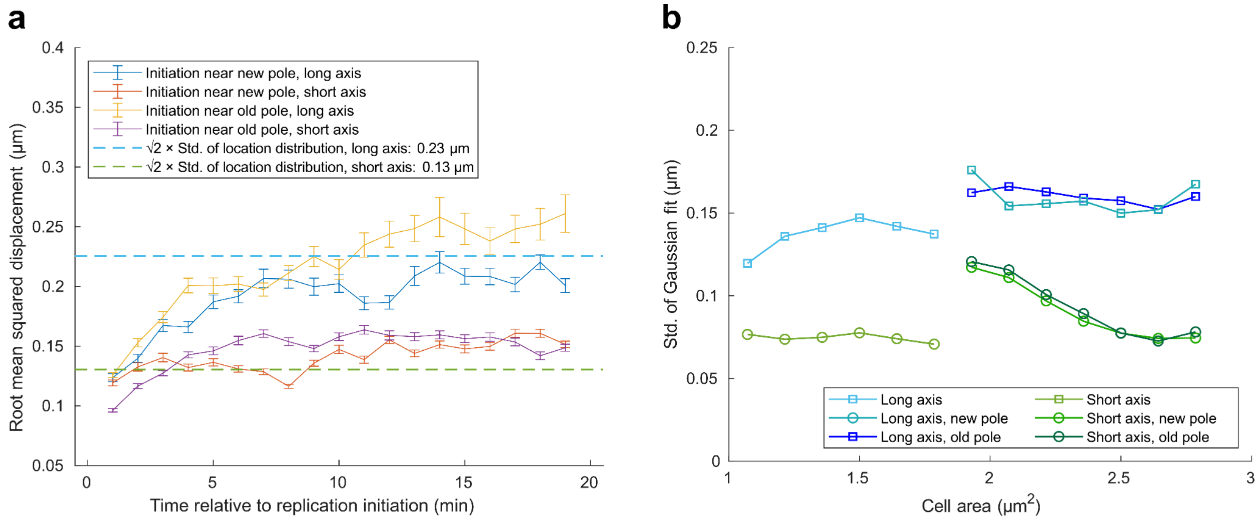

Fig. S3: Replisome RMSD following the replication initiation event. a, As in Fig. 2b. **b,** As in Supplementary Fig. S1b.

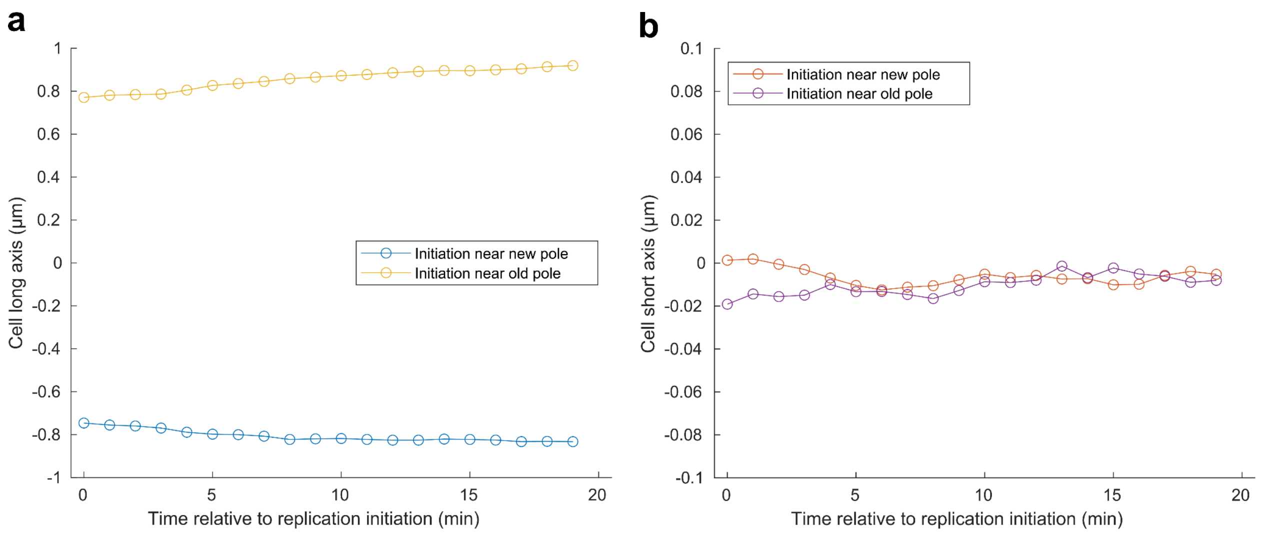

Fig. S4: Average replisome movement following the replication initiation event. Mean cell **a,** long axis and **b,** short axis coordinates of the replisome trajectories used for RMSD estimation in Fig. 2. For both the long and short axes 0 corresponds to the cell center.

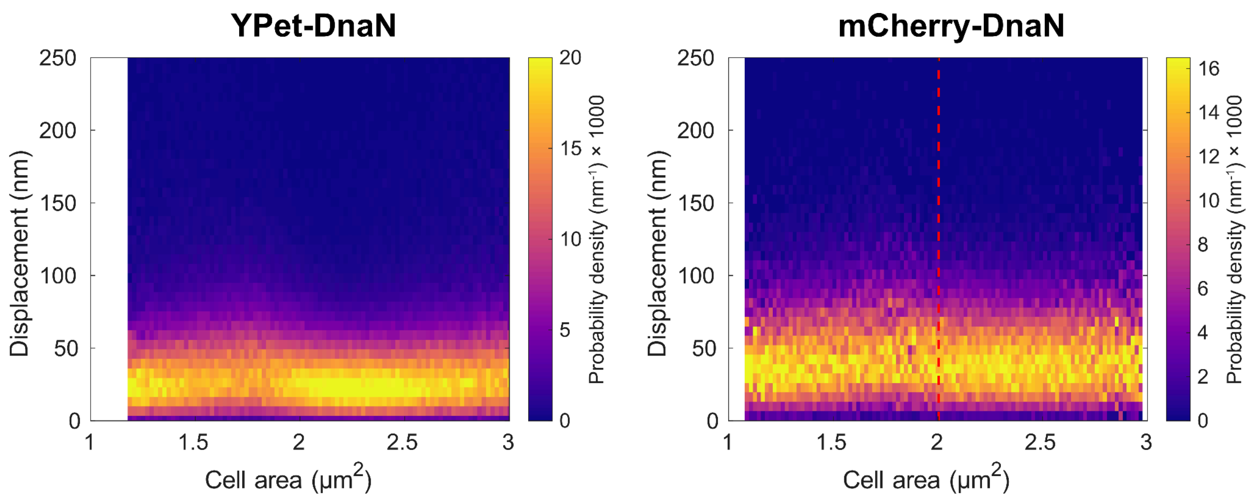

Fig. S5: Replisome displacements with different fluorescent protein fusions. (left) As Fig. 3b but for YPet-DnaN. (right) Data for mCherry-DnaN is the same as in Fig. 3b for comparison.

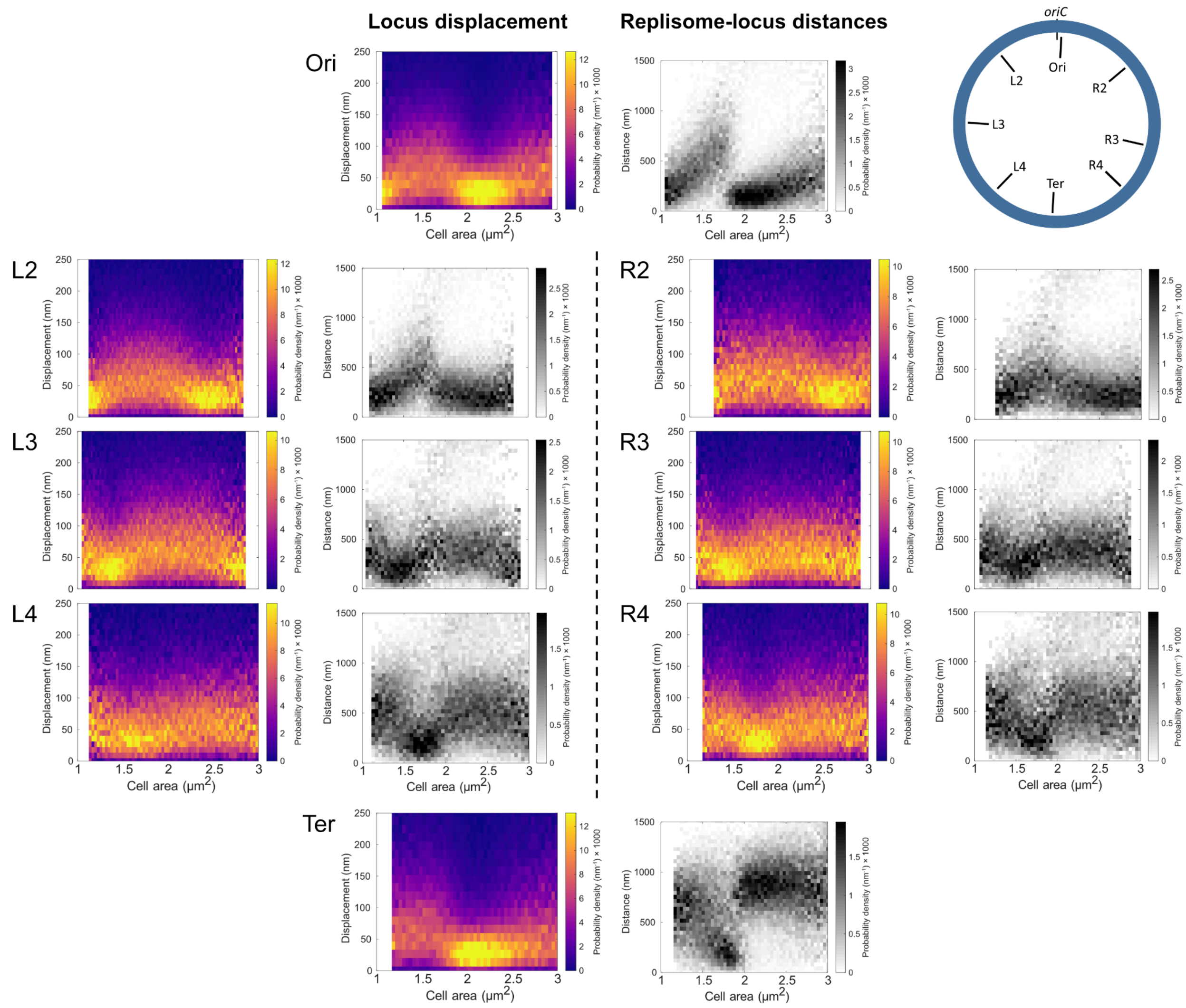

Fig. S6: Locus displacement minima coincide with replisome-locus colocalization. As Fig. 3a and 3d but for chromosome locus labels at different chromosomal positions relative to *oriC*.

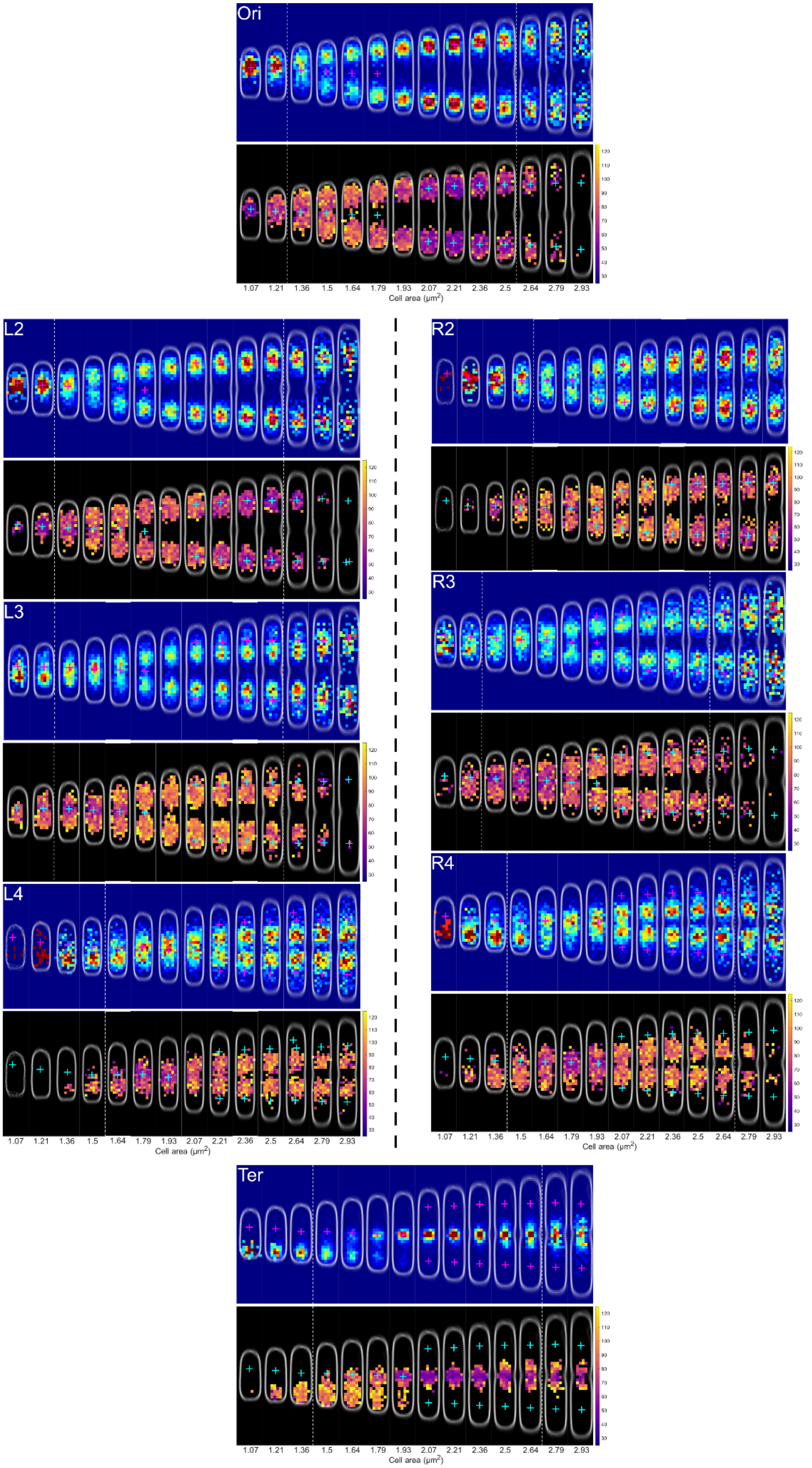

Fig. S7: Locus short-time-scale movement at different spatial positions. As Fig. 5b, but for a subset of loci.

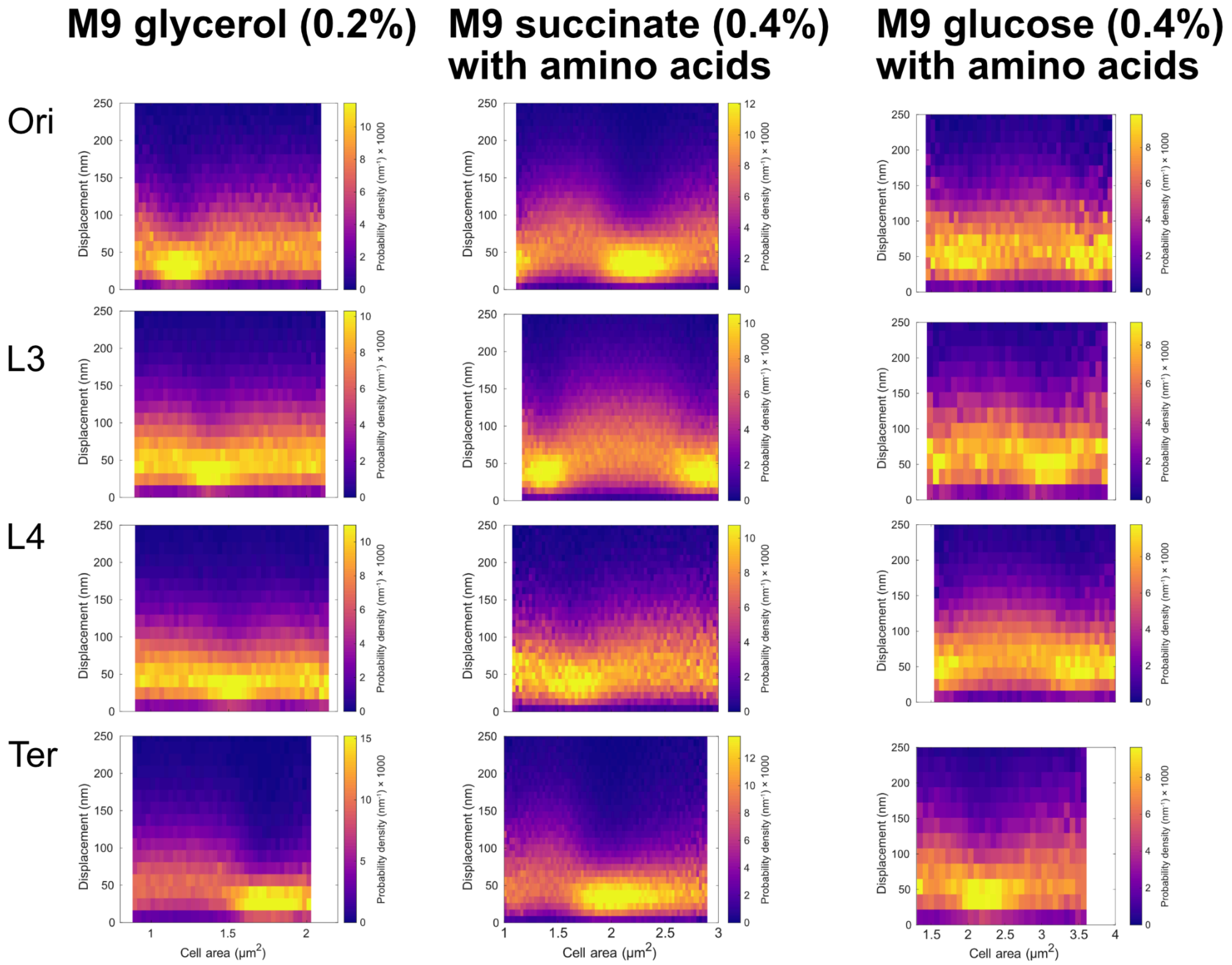

Fig. S8: Locus displacement minima at different growth conditions. As Fig. 3a (locus displacement) but for chromosome locus labels at different chromosomal positions relative to *oriC* in different growth conditions. Colors in heat maps as in Fig. 3a and c. The data for Locus ID: Ori, L3, L4 and Ter in M9 succinate (0.4%) with amino acids is the same as in Fig. 4.

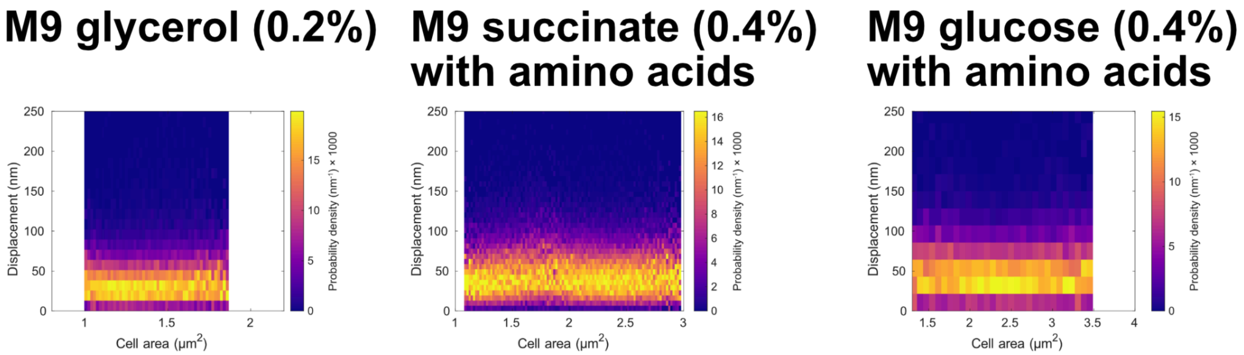

Fig. S9: Replisome displacements at different growth conditions. As Fig. 3b (replisome displacement) but for the replisome label mCherry-DnaN in different growth conditions. The colors in the heat maps as in Fig. 3b. The data for the replisome displacement heat map in M9 succinate (0.4%) with amino acids is the same as in Fig. 3b.

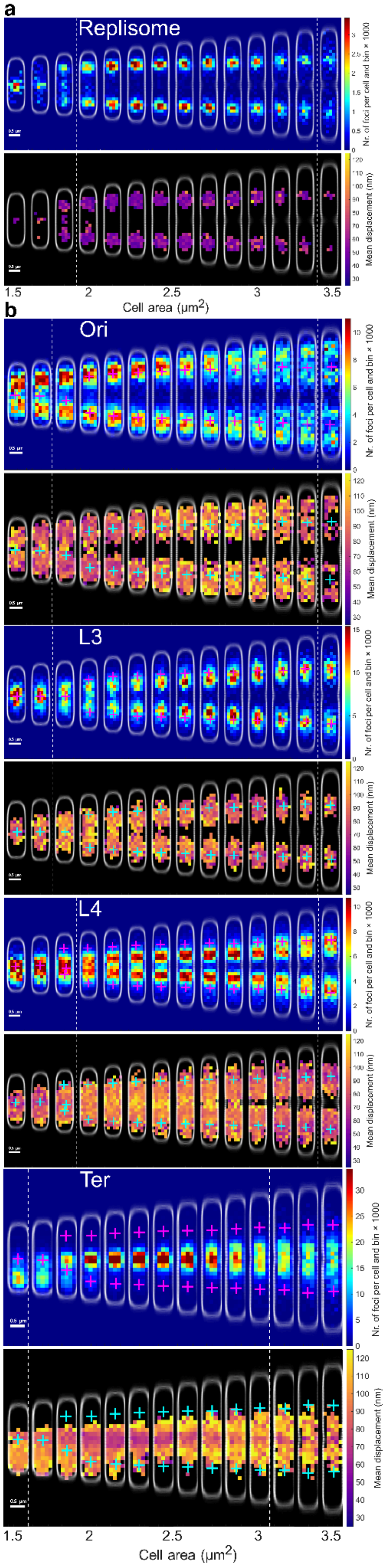

Fig. S10: Locus short-time-scale movement at faster growth rates. Two-dimensional histograms of fluorescent foci positions and displacements along the long and short axes of the cell as in Fig. 5, but for replisome and locus labeled strains at fast growth conditions. These experiments were performed twice, but only data from one replicate is shown

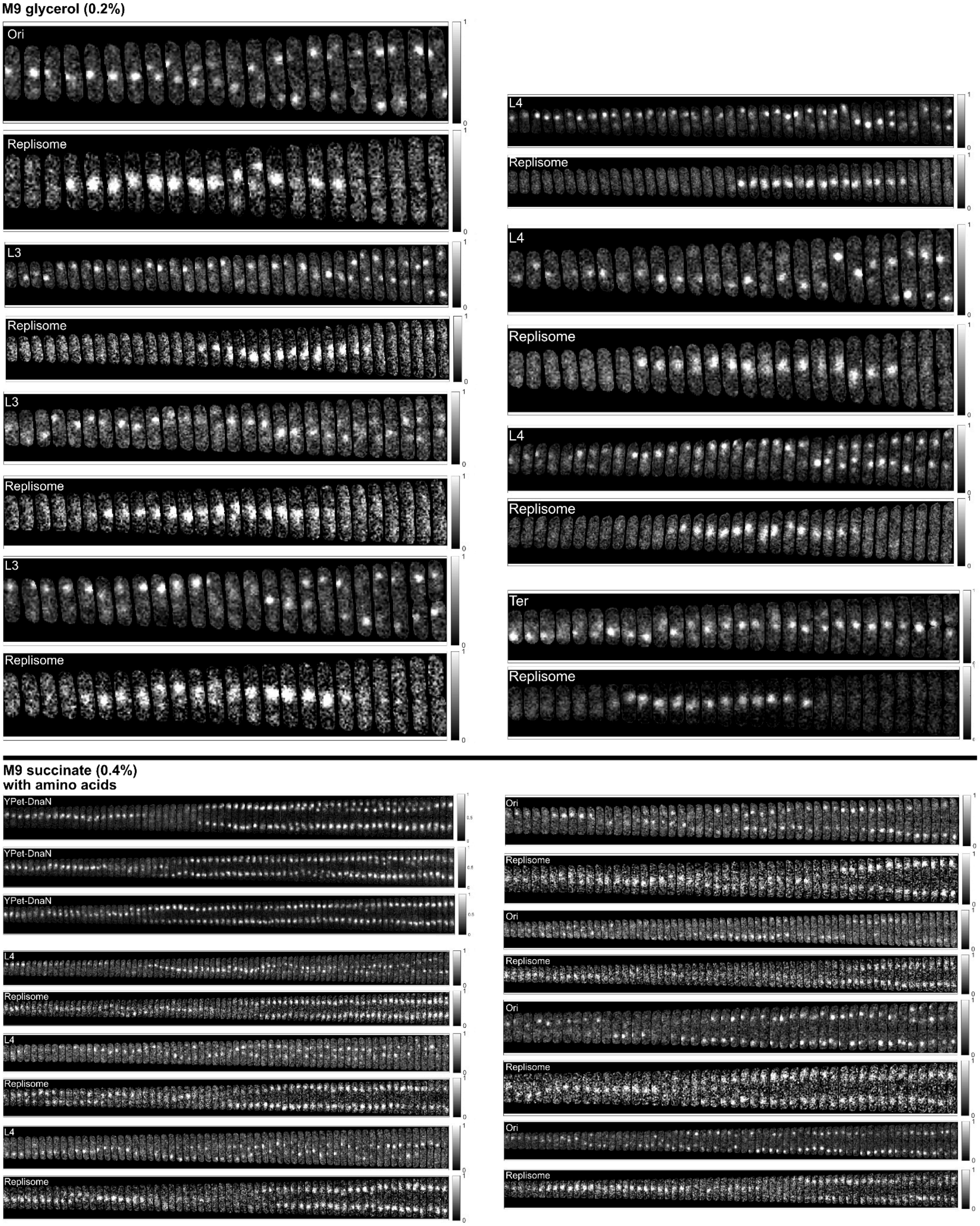

Fig. S11: Examples of single-cell tracks. Single-cell tracks of cells with the replisome and locus labels tracked over one generation in the indicated growth conditions. (Top) Single-cell tracks of strains with locus IDs: Ori, L3, L4 and Ter grown in slow growth conditions showing examples of the chromosomal and replisome labels from the same cells. (Bottom) Single-cell tracks of strains with the replisome label YPet-DnaN and locus IDs: Ori and L4 grown in intermediate growth conditions showing examples of the chromosomal and replisome labels from the same cells. Only the replisome tracks are shown for the strain with the YPet-DnaN label.

Table S1. Strain list. Table of strains used in this study. Genotypes of the strains with locus IDs indicate the base pair number where the chromosomal labels have been introduced.

Strain number	Strain	Genotype	Genomic distance to *oriC* (Mbp)	Reference
EL3495		*Eco MG1655 rph+ ΔmalI::frt intC::P59-malI-SYFP2-frt ΔgtrA::SpR mCherry-dnaN-frt*	NA	This study
**DerivaǮves of EL3495**				
EL4040	Locus ID: Ori	*3960236::MalOx12::KanR*	0.03	This study
EL4041	Locus ID: R2	*4569174::MalOx12::KanR*	0.6	This study
EL4043	Locus ID: R3	*609415::MalOx12::KanR*	1.4	This study
EL4044	Locus ID: R4	*1095536::MalOx12::KanR*	1.8	This study
EL4046	Locus ID: Ter	*1637533::MalOx12::KanR*	2.3	This study
EL4047	Locus ID: L4	*2228702::MalOx12::KanR*	1.7	This study
EL4048	Locus ID: L3	*2818831::MalOx12::KanR*	1.1	This study
EL4049	Locus ID: L2	*3418776::MalOx12::KanR*	0.5	This study
EL4050	Locus ID: R1	*4158275::MalOx12::KanR*	0.23	This study
EL4051	Locus ID: L1	*3720965::MalOx12::KanR*	0.25	This study
EL4092	Locus ID: R5	*1381955::MalOx12::KanR*	2.1	This study
EL4093	Locus ID: L5	*1893823::MalOx12::KanR*	2	This study
**Other strains**				
EL2290		*Eco BW25993 rph+ DELphi80 ΔydbL::parS-FRT-cat-FRT DELgtrA::P58-mCherry-parB-SpR kan-YPet-dnaN*	NA	Knöppel et al. (2023)

Supplementary References

1. Knöppel, A., Broström, O., Gras, K., Elf, J. & Fange, D. Regulatory elements coordinating initiation of chromosome replication to the *Escherichia coli* cell cycle. *Proc. Natl. Acad. Sci. U. S. A.* **120**, e2213795120 (2023).
