# Supplementary material for: The Escherichia coli chromosome moves to the replisome
Source: Nat Commun. 2024 Jul 17;15:6018. doi: 10.1038/s41467-024-50047-z (PMC11255300; doi:10.1038/s41467-024-50047-z)
Supplement: Supplementary file 3 — Description of Additional Supplementary Files [file 41467_2024_50047_MOESM3_ESM.pdf]

Description of Additional Supplementary Files:

Supplementary Dataset 1: Statistics for all experiments. Relevant statistics for all figures in this study.

Supplementary Dataset 2: Statistics of the two-dimensional histograms. The number of cells and fluorescent foci per cell area bin for selected two-dimensional histograms from each related figure
